# Long-range evolutionary constraints reveal *cis*-regulatory interactions on the human X chromosome

**DOI:** 10.1038/ncomms7904

**Published:** 2015-04-24

**Authors:** Magali Naville, Minaka Ishibashi, Marco Ferg, Hemant Bengani, Silke Rinkwitz, Monika Krecsmarik, Thomas A. Hawkins, Stephen W. Wilson, Elizabeth Manning, Chandra S. R. Chilamakuri, David I. Wilson, Alexandra Louis, F. Lucy Raymond, Sepand Rastegar, Uwe Strähle, Boris Lenhard, Laure Bally-Cuif, Veronica van Heyningen, David R. FitzPatrick, Thomas S. Becker, Hugues Roest Crollius

**Affiliations:** 1 Ecole Normale Supérieure, Institut de Biologie de l'ENS, IBENS, 46 rue d'Ulm, Paris F-75005, France; 2 CNRS, UMR 8197, Paris F-75005, France; 3 Inserm, U1024, Paris F-75005, France; 4 Brain and Mind Research Institute, Sydney Medical School, University of Sydney, Camperdown, New South Wales 2050, Australia; 5 Institute of Toxicology and Genetics and European Zebrafish Resource Centre, Karlsruhe Institute of Technology, Hermann-von-Helmholtz-Platz 1, 76344 Eggenstein-Leopoldshafen, Germany; 6 MRC Human Genetics Unit, MRC Institute of Medical Genetic and Molecular Medicine, University of Edinburgh, Edinburgh EH4 2XU, UK; 7 Paris-Saclay Institute for Neuroscience (Neuro-PSI), UMR9197 CNRS-Université Paris Sud, Avenue de la Terrasse, Gif-sur-Yvette 91190, France; 8 C.D.B. Division of Biosciences, Anatomy building, UCL, Gower street, London, WC1E 6BT, UK; 9 Department of Tumor Biology, The Norwegian Radium Hospital, 0310 Oslo, Norway; 10 University of Southampton and University Hospital Southampton NHS Foundation Trust, Centre for Human Development, Stem Cells and Regeneration, MP808, Faculty of Medicine, Southampton General Hospital, Tremona Road, Southampton 16 6YD, UK; 11 Cambridge Institute for Medical Research, University of Cambridge, Hills Road, Cambridge CB2 OXY, UK; 12 Institute of Clinical Sciences, MRC Clinical Sciences Centre, Faculty of Medicine, Imperial College London, Hammersmith Hospital Campus, Du Cane Road, London W12 0NN, UK; 13 Department of Clinical Medicine, University of Bergen, Bergen 5009, Norway

## Abstract

Enhancers can regulate the transcription of genes over long genomic distances. This is thought to lead to selection against genomic rearrangements within such regions that may disrupt this functional linkage. Here we test this concept experimentally using the human X chromosome. We describe a scoring method to identify evolutionary maintenance of linkage between conserved noncoding elements and neighbouring genes. Chromatin marks associated with enhancer function are strongly correlated with this linkage score. We test >1,000 putative enhancers by transgenesis assays in zebrafish to ascertain the identity of the target gene. The majority of active enhancers drive a transgenic expression in a pattern consistent with the known expression of a linked gene. These results show that evolutionary maintenance of linkage is a reliable predictor of an enhancer's function, and provide new information to discover the genetic basis of diseases caused by the mis-regulation of gene expression.

## Abstract (web summary)

Enhancers regulate the transcription of genes over long genomic distances. Here, the authors show that enhancer function is correlated with maintenance of linkage between non-coding elements and neighbouring genes in the human X chromosome and that enhancers in zebrafish drive expression in a pattern consistent with the expression of a linked gene.

## Graphical abstract

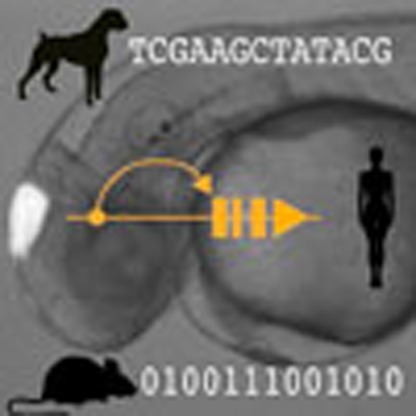

C*is*-regulation is a vital mechanism for the normal development and health of an organism. The *cis*-regulation of protein-coding gene expression in vertebrate genomes is mediated by regulatory factors binding to enhancer elements that may be located as much as 1.5 Mb from their target genes^1^,^2^, and longer distances are entirely possible. Given the importance of this *cis*-interaction, negative selection is thought to prevent the evolutionary fixation of rearrangements that would either physically dissociate the enhancer from the target gene or separate them by an excessive genomic distance. Genomic regions bearing these properties have been described as genome regulatory blocks^3^,^4^, but systematic efforts to exploit this evolutionary signature on a genomic scale^5^ have yet to be experimentally validated. Here we perform such an analysis on the human X chromosome, by developing a score that measures the evolutionary linkage between putative enhancers and their surrounding genes. We show that conserved noncoding elements (CNEs) showing the highest linkage scores are also enriched in functional marks such as epigenetic modifications characteristic of enhancers. We experimentally test >1,000 CNEs for their ability to replicate the expression pattern of their most strongly linked genes, and validate the predicted association for 60% of the cases where the expression pattern of the target gene was known. We finally show that putative enhancers linked to the same target gene are enriched in sequence motifs that may trigger the binding of specific transcription factors.

## Results

### Prediction of CNE/target gene associations

**Figure 1:**
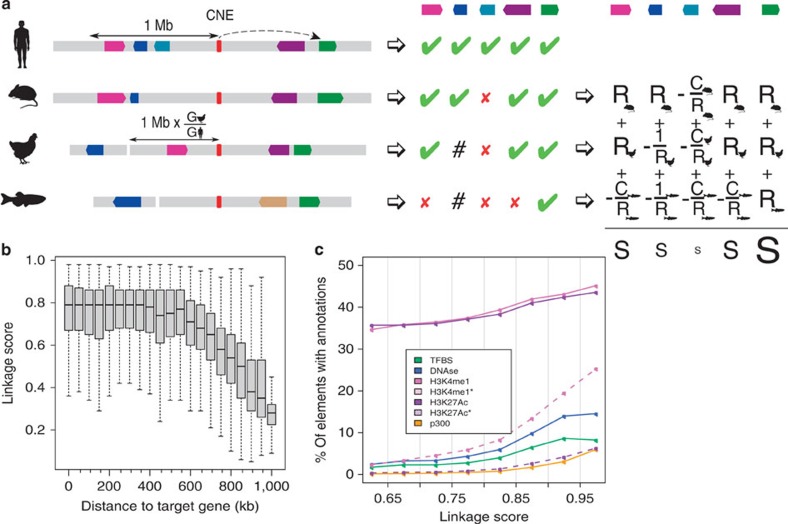
Scoring evolutionary linkage. (**a**) Strategy to compute the linkage score. The presence of human genes in a 1-Mb radius around a CNE are recorded, as well as the simultaneous presence/absence of their orthologs in the vicinity of the orthologous CNEs in different species (green ticks/red crosses, respectively, in the middle panel; hash signs indicate genes located beyond the 1 Mb threshold). The presence of an orthologue is weighted by the degree of conserved synteny *R* between this genome and the human genome, while the costs for the absence of a gene account for the sequencing coverage C of the genome. The final linkage score *S* is the sum of these weights in the different genomes where the CNE is present (right panel). The gene(s) showing the maximum linkage score to a given CNE is considered to be the most likely target. (**b**) The linkage score of the CNE-target predictions were grouped in bins according to the genomic distance between the CNE and its predicted target (*x* axis). The median linkage score of the distributions (*y* axis) is stable for genes located up to ∼600 kb from the RegHsa element. (**c**) The linkage score is strongly correlated with an enrichment in annotations linked to enhancer function. An asterisk indicates data generated during this project.

We identified human X-chromosome CNEs by scanning a multispecies genomic alignment encompassing 46 vertebrate genomes^6^ (Supplementary Fig. 1A), and looked for conserved regions, excluding exons and repeat sequences (Methods). This set was then merged with CNEs previously identified in eutherian mammals^7^. Together, these regions represent 174,473 distinct CNEs covering 4.4% of the human X chromosome, likely to represent most noncoding sequences under conservation. To test the hypothesis that functional interactions translate in physical linkage, we first devised a scoring procedure based on evolutionary conservation of linkage between a CNE and one of the human genes located within a radius of 1 Mb from the CNE. For a given CNE, the position of the orthologous CNEs were first sought in all the vertebrate genomes that align at this position. Next, the orthologs of the human genes found in the 1-Mb radius were also collected in all vertebrate genomes. Four situations may arise depending on whether and where the orthologous gene is present: (i) it too is linked to the orthologous CNE in the defined radius, (ii) it is located on the same chromosome but beyond the defined radius, (iii) it is located on a different chromosome and (iv) it is not annotated in the genome. In each genome, each situation was diagnosed and labelled with a score that accounts for the conservation of synteny between the human genome and the genome of interest, and the sequencing coverage of the latter (Fig. 1a and Methods). The maximum genomic interval allowed for linking the orthologous CNE and gene(s) was conservatively taken as 1 Mb but scaled in each genome depending on its relative size compared with the human genome. Together, this linkage and synteny information was used to compute an absolute score *S*_*A*_ between each CNE and each human gene within the 1 Mb radius (0<*S*_*A*_<1), reflecting the degree of linkage between them in vertebrate genomes (Fig. 1a and Methods). For each CNE, the best scoring genes were selected as plausible targets, with no minimal score threshold (Supplementary Data 1), and CNEs targeting the same genes were merged if their positions were <100 bp apart (Supplementary Fig. 1B). These merged CNEs are hereafter called RegHsa elements. We identified 102,647 RegHsa elements on the X chromosome with a mean size of 88 bp. Only 1% of RegHsas are not associated with a potential target gene (that is, their distance to the nearest human gene exceeds 1 Mb), 37.5% are associated with a single predicted target (single targets), and 61.5% are associated with several target genes with identical maximal score (multiple targets, not necessarily contiguous). Such multiple targets occur when evolutionarily neutral breakpoints have not yet dissociated the locus, some ‘bystander' genes may be captured in a genome regulatory block between an enhancer and its target gene^4^, or an enhancer may regulate several neighbouring genes. Of the 812 protein-coding genes annotated on the X chromosome, 389 were associated with at least one RegHsa element, while some genes, including *DIAP2*, *DMD* or *ODZ1*, are associated with >100 RegHsa elements. Of the RegHsa elements predicted to target a single gene, 60.7% target a gene that is not their direct neighbour. Interestingly, we observe a remarkably stable median linkage score in a 600-kb radius from the RegHsa element, with a sharp drop in linkage score values beyond this distance (Fig. 1b). Although enhancers are known to function beyond 600 kb, this result may indicate that factors such as the three-dimensional chromatin conformation or breakpoint frequencies may generally be unfavourable to long-range regulatory interactions beyond this distance.

### The linkage score is correlated with functional marks

If our method correctly reflects a functional association between enhancers and their target genes, we expect the linkage score *S*_*A*_ to correlate with functional annotations known to be associated with enhancers. To examine this, we annotated all CNEs that constitute RegHsa elements with functional signals known to be associated with enhancer function including chromatin accessibility by DNAseI assays, H3K4me1, H3K4me3, H3K27ac histone modifications and transcription factor-binding assays obtained from seven human cell lines^8^, as well as p300 signals from the mouse embryonic heart, forebrain, midbrain and limb^9^,^10^. Because the human X chromosome is known to harbour a high proportion of genes involved in cognitive functions and expressed in neural tissues^11^, we also performed H3K4me1, H3K27ac and p300 ChIP-on-chip experiments on human foetal brain and mouse E14.5, E16.5 and P0 developing brain tissues (Methods). When ranking CNEs and target gene associations by increasing the *S*_*A*_ score, we observe a pronounced enrichment in all functional annotations (Fig. 1c and Methods), with a fivefold increase in DNAse1 accessibility (average over seven human cell lines) and a striking 10.8-fold increase in H3K4me1 marks in human developing brain. Notably, the enrichment is not solely a consequence of the positive correlation between linkage score and conservation (Supplementary Fig. 2) because the result remains even when controlling for conservation (Supplementary Fig. 3). High scoring RegHsa elements (*S*_*A*_>0.9) are linked to genes showing a marked enrichment in gene ontology (GO) terms, notably those associated to neuronal cell body, axon guidance and synapse (Supplementary Table 1). Finally, the linkage score *S*_*A*_ strongly correlates with an enrichment of known transcription factor-binding motifs (Supplementary Fig. 4). Together, these results indicate that *cis*-interactions predicted only using evolutionary information are enriched in functional enhancers. Notably, this result is not limited to the X chromosome, because when we compute the *S*_*A*_ score on autosomes, they also show the same enrichment in functional annotations as a function of linkage score (Supplementary Fig. 5).

### Functional validation of predicted interactions

**Figure 2:**
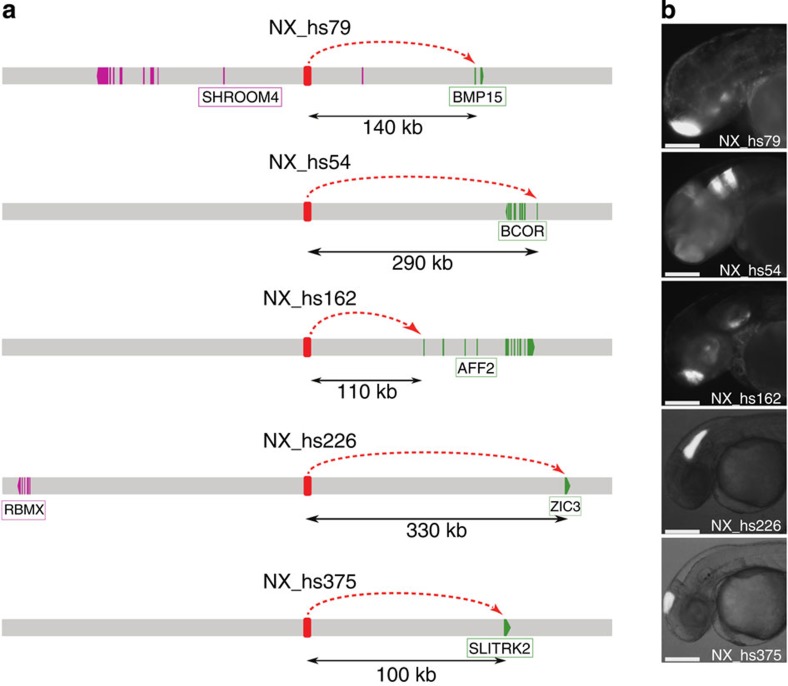
*Cis*-regulatory interactions predicted by the linkage score are experimentally tested in developing zebrafish. (**a**) Individual exons of the predicted target gene are depicted in green and of neighbouring genes in pink. The arrowhead indicates the direction of transcription. Distance in kilobases between the CNE and the promoter of the predicted gene are indicated. (**b**) The predictions are supported by transgenic analysis in zebrafish. Expression at 48 hpf: NX_hs79: telencephalon (scale bar, 125 μm); NX_hs54: hindbrain, telencephalon (scale bar, 125 μm); NX_hs162: telencephalon, hypothalamus, otic vesicle (scale bar, 125 μm); NX_hs226: hindbrain (scale bar, 200 μm); NX_hs375: midbrain (scale bar, 200 μm).

**Figure 3:**
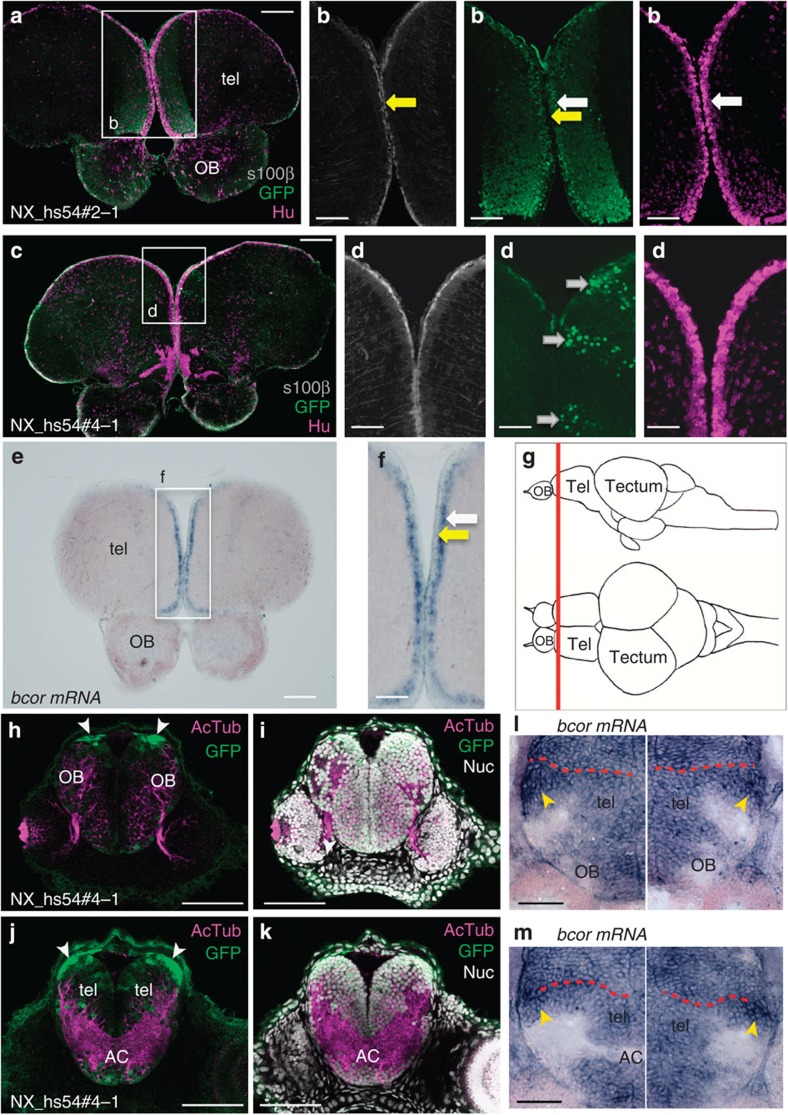
Neuroanatomical characterization of the element NX_hs54. This element includes RegHsa0032185 and was characterized in transgenic adult and juvenile zebrafish. (**a**–**d**) Immunohistochemical analysis of S100β (grey, radial glial stem cells), GFP (green), and Hu (magenta, neurons) expression in the telencephalon (level in **g**) in two different transgene integrations (2–1 and 4–1). Radial glial stem cells outline the telencephalic surface (yellow arrows, **b**) and generate neurons (white arrows, **b**)^40^. In one integration, GFP is expressed by virtually all neurons and their fibres underneath the radial glial cell layer (**b**). In the other integration (**c**,**d**), likely due to positional effects, GFP expression is restricted to individual neuronal clones (grey arrows). (**e**) *in situ* hybridization for endogenous *bcor* mRNA in the adult zebrafish telencephalon (level in **g**). *bcor* mRNA is expressed by the newborn neurons (white arrow, **f**) underlying the first cell layer of radial glial stem cells (yellow arrow, **f**). The extended GFP expression in transgenic lines is in agreement with GFP protein stability in neurons after endogenous *bcor* expression is switched off, and/or with the absence of a repressor element. (**g**) schematic lateral and dorsal views of an adult zebrafish brain showing the region (red line) examined in **a**,**c**,**e**.(**h**,**j**) Immunohistochemical characterization of juvenile GFP expression in NX_hs54#4-1 demonstrates overlap with endogenous *bcor* expression (**l**,**m**). Use of two anatomical markers: acetylated tubulin (**h,i**, **j,k**; magenta) and nuclear staining (**i**,**k**; greyscale) permits describing GFP expression in the telencephalon at two different section levels by confocal microscopy (**h** anterior to **j**). At 3dpf in NX_hs54#4-1 transgenic embryos GFP is widely expressed at a low level but also shows strong expression in the dorsal and lateral area adjacent to the ventricle (**h**,**j**; white arrowheads). This is similar to endogenous *bcor* mRNA, which also shows low level expression throughout the telencephalon and whole brain but has an area of strong expression next to the ventricle (**l**,**m**; yellow arrowheads, ventricle boundary marked by red dashed line). Abbreviations: AC, anterior commissure, tel, telencephalon, OB, olfactory bulb. Scale bars, **a**,**c**,**e**, 100 μm; **b**,**f**, 60 μm; d, 40 μm; **h**,**i**,**j**,**k**, 100 μm; **l**,**m**, 40 μm.

Next we directly tested the enhancer function of the interaction predicted by our comparative and functional genomic analyses by using transgenic assays. We selected 450 regions of ∼1 kb on the human X chromosome and overlapping 1,013 human RegHsa elements. These elements encompass a range of conservation levels and a large range of *S*_*A*_ scores (0.320–0.980) linking them to genes known to be involved in brain development (Supplementary Data 2). We examined their ability to drive specific green fluorescent protein (GFP) expression patterns in zebrafish embryos, by analysing at least five different insertions in F1 lines at 2 days post fertilization for each element. RegHsa elements with a reproducible or partially reproducible pattern of expression (448 cases) allowed us to test if the predicted target gene or genes of the enhancer are compatible with this pattern. For 323 RegHsas, expression data were available for the zebrafish (described in the ZFIN database^12^) for at least one predicted target. Of these, 200 RegHsa elements (60%) drive a transgenic GFP pattern that fully or partially overlaps the ZFIN pattern of one of the predicted targets (Fig. 2, Supplementary Figs 6 and 7, Supplementary Data 2 and Methods). These cases support the prediction that the enhancer indeed regulates the target gene showing the best *S*_*A*_ score. Consistent with this result, the average *S*_*A*_ score is significantly higher for the 200 supported enhancer–gene associations than for those that are not (*S*_*A*_ score 0.923 versus 0.863; *P*<2.10^−16^, Wilcoxon test). Interestingly, while 25% of tested RegHsa elements are conserved in zebrafish genomic DNA, this figure increases to 44% for elements with a predicted target that is supported in the transgenic experiments. This shows that conservation of a RegHsa elements is correlated with its functional property as enhancer, but it also shows that absence of conservation in fish does not preclude validation since 56% of enhancers are validated without conservation in fish. To further confirm the identity of the target gene in a limited number of cases, we verified if the enhancer drives GFP expression in the same brain region or cell type where the mRNA of its predicted target gene is expressed. To this end, we performed a detailed anatomical characterization of the GFP expression pattern in juvenile and/or adult zebrafish brains, for transgenic lines corresponding to 15 different human sequences elements overlapping 67 RegHsas (Methods). Out of the 15 transgenic assays analysed, 13 (87%) show that the gene that is evolutionarily linked to the RegHsa element is expressed in a pattern that completely (6 cases) or partially (7 cases) overlaps with the transgenic GFP pattern in either juvenile or adult zebrafish brain (Supplementary Data 3). For example, the RegHsa0032185 element is predicted to regulate the *BCOR* gene (*S*_*A*_=0.917) yet is located 286 Kb downstream of the nearest *BCOR* promoter (Fig. 2a). The elements reproducibly drive GFP expression in the developing zebrafish telencephalon and hindbrain. Neuroanatomical characterization of GFP expression in transgenic zebrafish lines carrying the RegHsa230032185 compared with endogenous zebrafish *bcor* mRNA expression in both juvenile and adult brains shows a strong overlap in the anterior telencephalon (Fig. 3). Critically, the GFP expression pattern strongly overlaps the endogenous zebrafish *bcor* mRNA expression (Fig. 3c). In addition, target gene predictions are consistent with published chromatin interaction maps. Indeed, of the 2,096 RegHsa elements that overlap the regions involved in 781 long-range chromatin interactions experimentally observed on the X chromosome by ChIA-PET in five human cell lines^13^, 69% are evolutionary associated (that is, show the best *S*_*A*_ score) with the same gene as shown to be involved in the chromatin interaction (*P* value <10^−5^, permutation test). Notably, this overlap is the same if we only consider cases where the predicted target is the nearest gene to the RegHsa element or if we consider cases where one or more genes separate the two. Together, these results support the original target gene prediction, which was obtained solely using genome comparisons. Interestingly, while our data agree with the ‘nearest gene' strategy 60% of the time (as does the ChIA-PET data, 62%), a greater rate of validation is observed when comparing our data with the ChIA-PET data (69%), which necessarily includes non-nearest genes.

### Motif discovery in CNEs assigned to the same target gene

**Figure 4:**
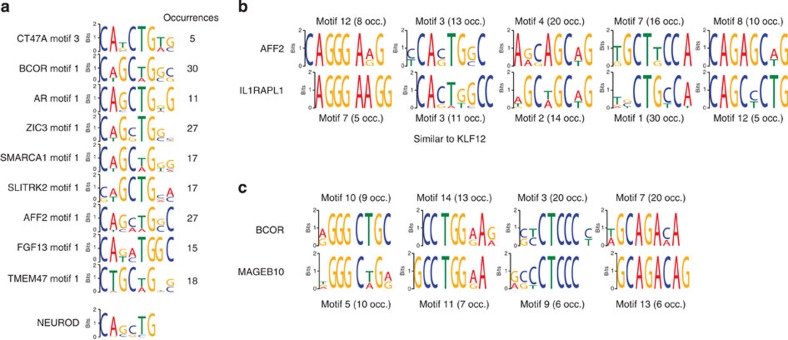
Motifs shared between RegHsa elements suggest co-regulated genes. (**a**) The NEUROD1/NEUROD2 binding site is recurrently found in multiple RegHsa elements linked to nine genes on the human X chromosome. (**b**) AFF2 and IL1RAPL1 share five overrepresented motifs in their linked RegHsa elements. Each motif logo is indicated together with the number of occurrences (occ.) in the set of RegHsa elements. Motif 3 is similar to the binding site of the KLF12 transcription factor. (**c**) BCOR and MAGEB10 share four overrepresented motifs in their linked RegHsa elements.

On average, 389 single target genes are associated with a mean of 17 RegHsa elements each with *S*_*A*_>0.9. We postulated that if different RegHsa elements are predicted to regulate the same target gene, they might share common sequence motifs recognized by the same transcription factor (TF). Consistent with this, we found significantly enriched motifs in elements targeting 124 genes (Methods), with up to 15 motifs per set of RegHsa targeting the same gene. Remarkably, different genes appear to be regulated by RegHsa elements that share the same motifs, despite the analysis being restricted to one human chromosome. The most striking case is a motif resembling the recognition sequence for the *NEUROD2* TF, present from 5 to 30 times in RegHsa elements targeting nine genes (Fig. 4a and Supplementary Data 4). *NEUROD2* is expressed in the developing brain and is important for lineage progression through chromatin remodelling^14^,^15^. Notably, several of the nine genes that are suggested here to be regulated by *NEUROD2* through common binding motifs are known to participate in different aspects of brain development and activity. In addition, 19 pairs of X-chromosome genes are linked to different sets of RegHsa elements that share three or more overrepresented motifs in common. For example RegHsa elements linked to *AFF2* and *IL1RAPL1* share five motifs in common (Fig. 4b), including a motif similar to that of the *KLF12* transcription factor, which is differentially expressed in a cellular model of neural progenitors^16^. Similarly, RegHsa elements linked to *BCOR* and *MAGEB10* share four overrepresented motifs (Fig. 4c) suggesting that each pair is co-regulated.

## Discussion

In summary, we describe a method to identify the evolutionary linkage between human CNEs (here, RegHsa elements) and neighbouring protein-coding target genes. We show that this linkage is indicative of a regulatory action of the element on the expression of the linked protein-coding gene. Some of these interactions were confirmed experimentally, but detailed characterization of the different CNEs is still required. Experimental methods are already able to indicate the interactions between enhancers and genes^8^,^13^,^17^,^18^,^19^ but they are strongly constrained by the tissue and time where and when the interaction takes place. In contrast, evolutionary linkage is independent of the tissue or time of expression of the gene, and is applicable to any sequenced vertebrate genome, as it was done here for human.

Regulatory mutations are known to cause diseases but few have been identified so far^20^,^21^,^22^,^23^, largely because the functional link between enhancers and their target gene is difficult to ascertain^24^. Here we provide a direct and simple approach to predict such interactions. For example, of the 45,449 RegHsa elements associated to one or more genes with a strong score (*S*_*A*_> 0.8), 8,217 elements target a gene where coding mutations have already been shown to cause intellectual disabilities. This strategy thus provides new material to accelerate the discovery of disease causing mutations.

## Methods

### Identification of CNEs

CNEs are defined based on their conservation in a range of vertebrate species, using an in-house algorithm called ‘ScanMaf' implemented in a python script (Supplementary Fig. 1a). ScanMaf scans the UCSC 46-species multiZ alignment and looks for conserved regions of a minimal length and identity, excluding exons annotated in Ensembl as well as repeats annotated by RepeatMasker and Tandem Repeat Finder. This algorithm does not require the presence of a fixed set of species in the alignment, but instead only requires a minimal number of seven species in addition to human, with no consideration of their respective phylogenetic group, allowing us to retrieve with the same procedure elements restricted to mammals as well as elements conserved between mammals and fish. It allows substitutions to occur, under a threshold of 12%, in each column of the alignment (in the minimal situation where only seven species are aligned to human, this threshold allows for one substitution); above this threshold columns are considered as conserved. The algorithm first identifies core windows of 10 bp containing at least 90% of such conserved columns. It then extends this nucleus in both the directions by allowing up to three non-conserved consecutive columns. If these human regions are conserved in the same subset of species, consecutive in each of their genomes, and separated by <100 bp in human, they were fused in a single resulting element in order to ease further analysis. These predictions were then fused with the regions obtained by the Siphy algorithm^7^. The resulting 174,473 distinct CNEs on the human X chromosome were used for further analysis. Each CNE was annotated with a score to characterize its evolutionary conservation between the human sequence and the other vertebrate sequences that align to this sequence. For this purpose, vertebrate genome sequences from the UCSC 46 species multiple alignments were classified into five groups according to their phylogenetic position: Boreoeutheria, Atlantogenata, Monotremes and Marsupials, Sauropsids and Amphibians, Teleostean fish. The maximum % ID between the human sequence and the sequences of each group, when present, are identified and summed to compute the conservation score. For example, a CNE is identified and is conserved from human to fish. The maximum % ID in each group are: Boreoeutheria 97% (with chimpanzee), Atlantogenata 68% (with elephant), Monotreme and Marsupials 62% (with opossum), Sauropsids and Amphibians 54% (with chicken) and Teleosts 49% (with medaka). The conservation score for this CNE will thus be: score=97+68+62+54+49=330.

### Scoring CNE-target genes evolutionary linkage

Families of orthologous genes were retrieved from the Ensembl database^25^ (version 66). Starting from the human genome as a reference (version hg19), the first step of the target prediction consists in collecting immediate neighbouring genes (distant from <1 Mb) of each given CNE within the human genome. A scoring procedure is then applied on these genes to try to identify the most probable CNE target. For any given CNE *i* present in *N* species, the absolute linkage score *S*_*Ai*_ is computed as follows:


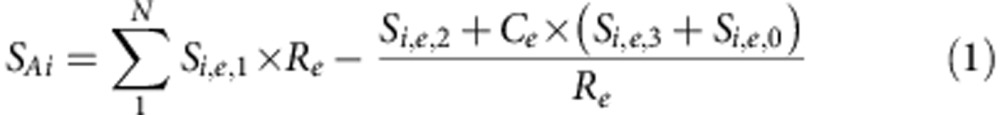


where *C*_*e*_ is a corrective factor to minimize the influence of genome assemblies obtained at low sequence coverage (Supplementary Table 2), *R*_*e*_ the rearrangement rate of the genome of species *e* by comparison with the human genome (see below and Supplementary Table 2) and *S*_*i*,*e*,0_, *S*_*i*,*e*,1_, *S*_*i*,*e*,2_, *S*_*i*,*e*,3_ the respective status of the orthologous gene considered in species *e*: *S*_*i*,*e*,0_ if absent (or mis-annotated), *S*_*i*,*e*,1_ if present and within distance *d* from the CNE, *S*_*i*,*e*,2_ if present and beyond distance *d* from the CNE, *S*_*i*,*e*,3_ if present but on another chromosome or scaffold. These *S*_*i*,*e*_ parameters take the value of 1 if the condition is fulfilled, 0 otherwise. Genome coverage, rearrangement rates and distance thresholds are listed in Supplementary Table 2. Distance *d* is taken as 1 Mb adjusted for the size of the genome of species *e* compared with the human (if the genome of *e* is 80% of the human genome, then *d*=0.8 Mb). The level of synteny *R*_*e*_ is computed as follows:


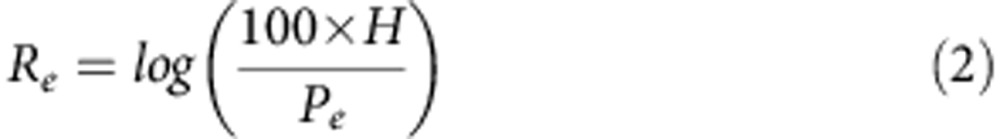


where *H* is the total number of gene pairs in the human genome, and *P*_*e*_ the number of these gene pairs that are direct neighbours (in conserved synteny) in species *e* with the human gene pairs. *R*_*e*_ thus varies between 0 (a genome with no gene pairs in conserved synteny) and 1 (the human genome against itself). Of note, the baboon (papHam1) and the lamprey (petMar1) genome sequences, despite being present in the 46-species multiple alignment, were not used for the target search because of the high degree of fragmentation of their assemblies. These linkage scores, after being calculated for every gene families neighbouring each CNEs, are then normalized in a [0,1] interval using a sigmoid transformation as follows:


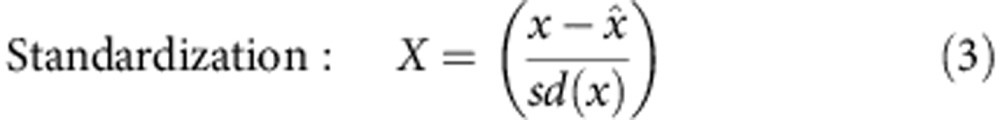



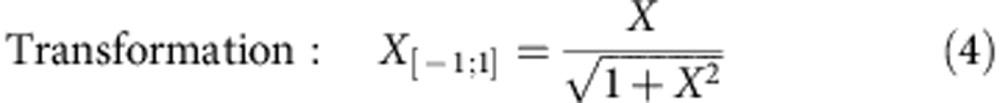



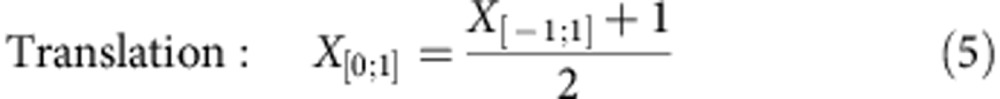


After sorting linked genes by descending linkage score, a relative score can be computed for each, corresponding to the linkage score difference between the top-ranking linkage score and the second-best linkage score. The greater the relative linkage score, the more contrast. However, if a CNE presents only one putative target in its environment, the corresponding gene family will have no relative score attributed. The relative score is useful to identify cases where, among all possible targets within 1 Mb of a given CNE, one gene stands out: this gene will have a high relative score, because there will be a high difference between its linkage score and that of the next-best target. CNEs targeting the same genes and located <100 bp apart were fused, resulting in 102,647 RegHsa elements. The complete set of RegHsa elements together with their scores and target genes are available in Supplementary Data 1. RegHsa elements linked to their target gene with a score>0.9 are available in a graphical interactive server on http://www.genomicus.biologie.ens.fr/genomicus.

### Enrichment in enhancer functional data

Functional information was collected from the Ensembl project for DNase hypersensitive sites (DHS)^26^, chromatin immunoprecipitation sequencing (ChIP-seq) for TFs^26^, and H3K4me1, H3K4me3 and H3K27ac histones modifications^26^ for seven different cell lines (Gm12878, H1-hESC, HSMM, HUVEC, K562, NHEK, NHLF, HMEC and NH-A). We also collected published p300 functional annotations for mouse developing heart^9^ and mouse developing forebrain, midbrain and limb^10^ (see below for links to public data sources). Finally, we generated p300, H3K4me1 and H3K27ac annotations using ChIP-on-chip on the human X chromosome with chromatin isolated from human fetal brain and E14.5 and P0 developing mouse brain (Methods). To compute the intersection between the functional data listed above and CNE intervals, the positions of the functional annotations and of the CNEs were compared. When the intervals overlapped by at least 1 bp, the CNE was assigned a ‘functional score' corresponding to the value of the overlapping signal weighted by the percentage of the CNE covered by the signal. For instance, if a 100-bp CNE overlaps a DHS peak of value 12 over 40 bp, the DHS value associated to the CNE is: 12 × (40/100)=4.8. For CNEs overlapping several distinct peaks, the resulting signal value is additive. In Fig. 1b and Supplementary Fig. 5, the proportion of RegHsa elements that overlap a functional annotation (with value >0) through at least one of their constitutive CNEs was computed for each of the annotations, for classes of RegHsa elements of increasing linkage score. To associate GO^27^ terms with X-chromosome genes predicted to be functionally linked to RegHsa elements, we used the PathwayStudio platform (Elsevier B.V., Amsterdam). GO annotations from lists of genes linked to CNEs above a certain linkage score thresholds were compared with the lists drawn from the complete list of genes of the X chromosome (Supplementary Table 1). Statistical significance was estimated by Fisher's test, without correction for multiple testing.

### Sources of public data for enhancer enrichment tests

CNEs were annotated with a range of functional annotations, both published and obtained in the course of this project:

ENCODE (Feb. 2012) DHS^26^.

(http://genome-euro.ucsc.edu/cgi-bin/hgTrackUi?hgsid=195751083&c=chr21&g=wgEncodeAwgDnaseUniform)

ENCODE (Feb. 2012) ChIP-seq for Transcription Factors^26^.

(http://genome-euro.ucsc.edu/cgi-bin/hgTrackUi?hgsid=195751457&c=chr21&g=wgEncodeAwgTfbsUniform)

ENCODE (Feb. 2012) H3K4me1, H3K4me3 and H3K27ac histones modifications^26^.

(http://genome-euro.ucsc.edu/cgi-bin/hgTrackUi?hgsid=195751457&c=chr21&g=wgEncodeBroadHistone).

In the 3 ENCODE data sets above, peaks correspond to local maxima of the different signals. We used data obtained in seven different cell lines (Gm12878, H1-hESC, HSMM, HUVEC, K562, NHEK, NHLF, HMEC and NH-A), by computing the mean of each functional signal in 25-bp windows along the X chromosome before intersecting these annotations with the CNE intervals.

Blow *et al*.^9^: p300 ChIP-seq data from mouse developing heart

(*http://www.nature.com/ng/journal/v42/n9/extref/ng.650-S2.xls*)

Visel *et al*.^10^: p300 ChIP-seq data from mouse developing forebrain, midbrain and limb.

(*http://www.nature.com/nature/journal/v457/n7231/extref/nature07730-s2.xls*)

### Overlap between this study and interactions shown by ChIA-PET

The genomic positions of RegHsa elements were compared with the regions shown by Li *et al*.^13^ to interact with gene promoters via ChiA-PET experiments. The best scoring genes of each overlapping RegHsa elements were compared with the genes that interact with the corresponding region by ChIA-PET. The two ‘experiments' (linkage score in this study and ChIA-PET by Li *et al*.^13^) were considered consistent if one of the linked genes (with maximal score) was the same as one of the gene shown to interact by ChIA-PET. Of the 102,647 RegHsa elements identified on the human X chromosome, 2,096 elements overlap regions shown in the ChIA-PET experiment to interact with a promoter. We compared the genes evolutionarily linked (with a maximum score) with these elements, and the gene(s) shown to interact, via their promoter, with the overlapping regions via ChIA-PET. For 1,454 elements (69%), the linked genes and the interacting gene are consistent. To compute a *P* value expressing the probability of obtaining the same result by chance, we performed 10.000 resamplings of the genes linked to the 2,096 RegHsa elements that overlap ChIA-PET enhancers. In each resampling, each RegHsa element was associated to the same number of best scoring linked genes, but randomly selected among all genes present in a 2-Mb window centred on the RegHsa element. If the ChIA-PET gene target was found among these randomly associated genes, we considered the two experiments to be consistent by chance. No resampling trial reached the number of coincidences between ChIA-PET and ‘linkage score' experiment obtained from in the real data. We thus estimate that the *P* value of the test is <10^−5^.

### ChIP-on-chip from human and mouse developing brain

This assay was performed for p300, H3K4me1 and H3K27ac as described^28^, with several modifications. Embryonic brain was isolated from human (three samples at 50 days of gestation) and mouse (E 14.5 and P0) embryos. Human fetal brain tissues were collected with informed written consent and ethical approval by Southampton and South West Hants LREC. Pools of whole brain were treated with 1.5% formaldehyde for 10 min at room temperature. Crosslinking was stopped by the addition of glycine to a final concentration of 0.125 M. The brain tissue was chopped into small pieces (∼1 mm^3^) with a razor blade in cold 1 × PBS and single cell suspension was made using dounce homogenizer. The cells were swelled on ice for 10 min. in 25 mM HEPES, pH 7.8, 1.5 mM MgCl_2_, 10 mM KCl, 0.1% NP-40, 1 mM DTT (dithiothreitol) and protease inhibitor cocktail (Roche) and the nuclei were collected by centrifugation at 2,500 r.p.m. Nuclei were resuspended in ‘sonication buffer' containing 50 mM HEPES pH 7.9, 140 mM NaCl, 1 mM EDTA, 1% Triton X-100, 0.1% Na-deoxycholate, 0.1% SDS and protease inhibitors, and sonicated on ice to an average length of 200–500 bp. The samples were centrifuged at 14,000 r.p.m. and the chromatin was precleared with protein-A-Dynabeads. Precleared chromatin were imunoprecipitated with 5 μg of H3K4me1 (ab8895, Abcam), 5μg of H3K27Ac (ab4729, Abcam) and 10 μg of p300(C-20:sc585,Santacruz) antibodies and the immune complexes were collected by incubating with protein-A-Dynabeads. The beads were washed twice with ‘sonication buffer', twice with sonication buffer containing 500 mM NaCl, twice with 20 mM Tris, pH 8.0, 1 mM EDTA, 250 mM LiCl, 0.5% NP-40, 0.5% Na-deoxycholate and twice with TE buffer. The immunocomplexes were eluted with 50 mM Tris, pH 8.0, 1 mM EDTA and 1% SDS at 65 °C for 10 min., adjusted to 200 mM NaCl and incubated at 65 °C overnight to reverse the cross-links. After successive treatments with 10 μg ml^−1^ Rnase A and 20 μg ml^−1^ proteinase-K, the samples were eluted into 50 μl H2O using the QIAquick Spin Gel Purification Kit (Qiagen). ChIP DNA and input DNA were labelled with Cy5 or Cy3, respectively, using random priming with dye-labelled random hexamers and hybridized according to the manufacturer's protocol to a HX1 (2.16 million probes) custom microarray containing specific tiled regions encompassing 99.2 and 93.8 Mb of the human and mouse X chromosome, respectively, (Nimblegen). Arrays were scanned on a NimbleGen MS 200 Microarray scanner (Nimblegen) using a laser power of 100% and 2-μm resolution and TIFF images analysed using MS 200 Data Collection software to quantitate raw signal intensities. Computational analysis of the data was carried out using the Ringo R/Bioconductor package^29^.The Cy5/Cy3 log_2_ ratio were calculated for each probe and scaled by subtracting Tukey's biweight mean, as recommended in the standard manufacturer's procedure (Nimblegen). Before calling ChIP-enriched regions, we performed a smoothing over individual probe intensities. ChIP-enriched regions were called using the findChersOnSmoothed function from the Ringo package, using parameters distCutOff=100 and minProbesInRow=6. ChIP-chip data have been deposited to the GEO repository under accession number GSE57358. Human fetal tissue was obtained with informed consent and according to the protocol ethically approved by Southampton and South West Hants LREC. The principal investigator of these ethical approvals is D.I.W.

### Zebrafish transgenic assays of human REG elements

Sequences chosen for testing were PCR-amplified from human genomic DNA as elements of 1–3 kb size and subcloned into pCR8 plasmid to create an entry vector for the Gateway system. Subsequent cloning into a Tol2-GFP-destination vector, microinjection of the plasmid into fertilized zebrafish eggs as well as fluorescent screening of the embryos, establishing transgenic lines and expression pattern documentation have been described elsewhere^30^. All the experiments were approved by the animal ethics committee of the University of Sydney and in accordance with the German protection standards and were approved by the Government of Baden-Württemberg Regierungspräsidum Karlsruhe, Germany

### CNE-target gene predictions and transgenic experiments

Transgenic elements tested in the course of this study were chosen based on a number of criteria, including sequence conservation, location near genes of medical interest and published information on enhancer function. Importantly, they were never chosen based on the linkage score described in the Methods section 2. It is therefore possible to use the transgenic experiments as a means to provide an indirect support for the two predictions:

1. The regulatory potential of the CNE, if the latter drives specific and reproducible expression of the reporter gene (GFP) during zebrafish development.
2. The target gene being regulated by the CNE, if the GFP expression pattern overlaps the expression pattern of the predicted target.

The experiment may fail to deliver an interpretable result independently of the absence of function of the CNE as human regulatory enhancer. For example, this may happen if the CNE regulates the expression of its target genes exclusively after zebrafish development is complete, if the reporter cassette (see Methods section 6) is integrated in repressive chromatin environment, or if the human sequence element is not recognized by the zebrafish orthologue of the human TF (for example, if the zebrafish ortholog has an affinity for a different sequence, or if it is altogether absent from the zebrafish genome).

Here 436 human sequence elements were tested using zebrafish transgenic experiments (Methods). These sequence elements include 1,013 RegHsa elements (Supplementary Data 2). Thereafter, results will be described and discussed in terms of RegHsa elements, because RegHsa elements are the basic ‘units' of human sequences that are linked to target genes using the *S*_*A*_ score described in Methods. Of the 1,013 RegHsa, 574 (57%) overlap sequences that produced inconsistent expression patterns in the different F1 lines or no expression at all. The remaining 448 RegHsa produced partially or fully consistent GFP expression patterns and were further exploited. Of these, 125 elements are evolutionarily linked to one or several human genes with orthologues in zebrafish that have no recorded expression pattern in the ZFIN database. Therefore, these elements are not useful to assess the prediction that the RegHsa element is an enhancer that regulates its linked gene(s). Only the remaining 323 RegHsa elements fulfil the two conditions required to test if the transgenic experiment supports the prediction: they are contained in a sequence element that drives a partially reproducible or reproducible GFP pattern during zebrafish development, and their predicted human gene target(s) include at least one human gene with a zebrafish orthologue of known expression pattern. For the transgenic experiment, we examined the GFP expression pattern in at least five independent zebrafish F1 lines to assess the reproducibility of the pattern. The pattern was then manually recorded using ZFIN nomenclature according to the tissue(s) showing GFP expression. For the known expression pattern of the zebrafish orthologue(s), we listed the tissue(s) showing expression by *in situ* hybridization during development, or the tissue(s) affected by a mutation in the gene, or both (ZFIN database: http://zfin.org). The GFP expression patterns and the ZFIN expression patterns were then compared, and results show that 200 RegHsa elements (60% of 323) drive a GFP expression pattern in a tissue that is included in the published expression pattern of the predicted target, or of one of the predicted targets when several exist with an identical maximum linkage score. A schematic diagram of the decision process described here is shown in Supplementary Fig. 6. We tested the possibility that this result may be due to a bias in the RegHsa elements. Indeed, the probability of a RegHsa elements driving GFP expression by chance in at least one tissue in common with its predicted targets increases in proportion to the number of predicted targets. However, the 200 RegHsa elements that drive a GFP pattern that overlaps with the pattern of a target gene possess an average of 4.7 targets, while the 123 RegHsa elements that drive a GFP pattern that does not overlap with that of any of the target genes possess an average of 6.3 targets. Therefore the results are consistent with the starting hypothesis, that a strong evolutionary linkage score between a CNE and one or more neighbouring genes reflects a regulatory role of the CNE on the expression of one of the linked genes.

### Anatomical characterization of zebrafish GFP expression

(a) Adult GFP expression analysis: the dissected brains of F1 adult (3–9 months) zebrafish from two different transgene integrations of each tested element were fixed in 4% paraformaldehyde for 4 h at room temperature. The following primary antibodies were applied onto free-floating 80-μm-thick vibratome sections: GFP (1:500, chicken, Aves Laboratories), HuC/D (1:2,000, human, a gift from Dr B. Zalc, Salpêtrière Hospital, Paris), glutamine-synthase (1:500, mouse, Millipore). DAPI (diamidino-2-phenylindole; 1:3,000) was used as a nuclear counterstain. Secondary antibodies raised in goat coupled to AlexaFluor dyes (Invitrogen) were used (1:1,000). HuC/D as a neuronal marker and glutamine-synthase as a glial marker label the two main cell types of the zebrafish telencephalon^31^ and therefore make it possible to identify GFP expressing cells. All images were taken on a Zeiss LSM700 confocal microscope using × 20 air, × 40 oil or × 63 oil objectives. Images were processed using the ZEN software (Zeiss). Composite images were automatically stitched upon acquisition using ‘Tilescan' mode on the Zeiss ZEN software. (b) Adult mRNA expression analysis by chromogenic *in situ* hybridization: the dissected brains of adult (3–9 months) zebrafish from the wild-type AB strain were fixed in 4% paraformaldehyde for 14 h at 4 °C. Whole brains were incubated at 65 °C for 18 h in 2 ng μl^−1^ digoxigenin (DIG)-labelled mRNA probes. After hybridization, the brains were embedded in 3% agarose and 80-μm-thick cross sections were cut using a vibratome. The sections were blocked in blocking buffer (2% normal goat serum, 2 mg ml^−1^ bovine serum albumin) and incubated with anti-DIG AP Fab fragments (sheep, Roche, 1:5,000) and the signal was revealed with NBT/BCIP. Pictures were taken on a Nikon AZ100 microscope equipped with a Nikon DS Ri1 camera. Expression of GFP from transgenic lines and the expression of mRNA in wild-type fish were compared manually using neuroanatomical landmarks and immunohistochemical labels. (c) Detailed expression analysis in juvenile fish: F1 juvenile zebrafish (3dpf and 6dpf) from three different transgene integrations of each tested element were anaesthetised in MS-222 and fixed immersion in 4% paraformaldehyde in 4% sucrose PBS (pH7.3). Samples were split into two sets. One set (called neuroanatomy test) was examined using wholemount immunohistochemistry to detect GFP in the context of two immunohistochemical neuroanatomical markers: SV2 and acetylated α tubulin. These neuroanatomical markers provide well characterized neuroanatomical landmarks to interpret the location of GFP expression. The protocol followed was the same as that employed to prepare samples for zebrafishbrain.org^32^. The second set (called *in situ* test) was used to perform wholemount fluorescent *in situ* hybridization using DIG-labelled probes and tyramide detection according to the protocol of Lauter *et al*.^33^ followed by immunohistochemical detection of GFP. Both the sets of samples were examined using confocal microscopy from a dorsal and lateral aspect (eye removed). Stacks were examined in 3D using Fiji software for neuroanatomical location and overlap between native gene expression and GFP expression. Frequently the *in situ* test set showed poor expression data for the *in situ* hybridization channel. For these sets, *in situ* hybridization was carried out on wild-type AB embryos using chromogenic detection of DIG-labelled probes according the standard protocol of the Thisse laboratory^34^. Expression could then be compared between this sample and the neuroanatomical test sample. Output data took the form of text annotations of the neuroanatomical locations of GFP expression and its comparison with native zebrafish gene expression.

### Analysis of sequence motifs in RegHsa elements

(a) *De novo* motif identification in CNEs. Conserved motifs were searched in each set of CNEs constitutive of a given REG element as long as they fulfil the following conditions (to minimize false positives): they must be associated to a single best target gene with a linkage score >0.3 and a relative score >0.05. Only sets comprising at least 10 CNEs (153 sets in total) were searched for possible motif enrichment. Motifs were detected using MEME3 (ref. 35) with the following options and parameters: -dna -nmotifs 15 -revcomp -mod anr -wg 6 -ws 1 -minsites 5 -maxw 8. The different motif occurrences identified by MEME in the CNEs were further reviewed to increase the motif stringency. This was done by removing sequences presenting <80% identity with the first motif occurrence identified by MEME, which is considered to be the most similar to the motif. A threshold score characterizing each motif is then defined as the lowest weight obtained while matching the motif against each of its constitutive sequences, using the matrix-scan program of the RSATools suite^36^. This score will be used to seek the motif in other control CNE sets. For all RSAT tools used here, the background option (‘-bgfile') was applied, with background statistics calculated on the entire set of CNEs using the oligo-analysis program with the following parameters: -l 2 -1str –return freq. This program thus determined the frequencies of every possible dinucleotide in the total set of CNEs, and used these as background frequencies to compute the significance of observed motifs. (b) Are the motifs significantly overrepresented? Two statistical tests are further applied to eliminate motifs that may be due to chance occurrence. The first test consists in calculating a *P* value associated to the number of motif hits observed in the CNE set, by searching the motif in 1,000 random sets comprising the same number of CNEs, using matrix-scan and the weight threshold value previously computed. This *P* value reflects the number of times an equal or higher number of motif occurrences are found by chance, compared with the set of CNEs predicted to target the same gene. The second test consists in the search for motifs in the same CNE set but using shuffled motifs. These shuffled motifs are obtained by a column permutation of the motif of interest (reference motif), repeated up to 1,000 times until we obtain up to 10 motifs that are significantly different from the reference motif and from each other (the Pearson coefficient of correlation between position weight matrices, obtained by RSAT compare-matrices must be <0.30). Motifs were ultimately considered significant with this second test if none of the shuffled matrices found >2/3 of the number of matches found by the original motif, in the same CNE set. (c) Comparing motifs between sets of CNEs: after this filtering step, motifs obtained for distinct sets of CNEs targeting different genes were compared using the RSAT compare-matrices program^36^. Two motifs were considered as similar if the Pearson coefficient of correlation between their position weight matrices, further weighted by the length of the match, was >800. (d) Are CNEs enriched in known motifs? We computed the proportion of CNEs that match known motifs, as a function of increasing evolutionary linkage score to a neighbouring gene (similar to Fig. 1b). CNEs were divided in classes of increasing linkage score, and each class was compared with the TRANSFAC database (complete vertebrate motifs; version 2010)^37^, to a list of sites established by high throughput SELEX^38^ and to matrices from the JASPAR database (version 2011)^39^ (Supplementary Fig. 4). Matches between CNEs and matrices of known motifs were identified using the matrix-scan program from RSAT^36^, with the background as described above and with the following parameters: -1str –lth score 5.0. Only motifs showing a score >15 were considered. A full description of motifs shown in Fig. 4 is in Supplementary Data 4.

### Code availability

Python scripts to identify CNEs in multiple alignments and to compute the linkage score are freely available under a GNU GPL v3 or later, and under a CeCiLL v2 license in France, as a GitHub project named Regulus: https://github.com/DyogenIBENS/Regulus.

## Author contributions

M.N. and H.R.C designed the evolutionary genomics method and M.N. performed analyses with help from A.L.. M.I., M.F., E.M. S.Ri. and S.Ra. performed zebrafish transgenic experiments. H.B. performed ChIP-chip experiments. M.K. and T.A.H. performed zebrafish *in situ* experiments. C.S.R.C analysed ChIP-chip data. D.I.W. provided human fetal tissues. D.R.F, V.V.H, S.W., B.L., U.S., L.B.-C., T.S.B., H.R.C. co-led the project with advice from F.L.R. T.S.B. designed the initial study. M.N. and H.R.C. wrote the manuscript with contributions from L.B.-C., M.K., T.A.H, U.S., B.L., V.V.H., T.S.B. and D.R.F.

## Additional information

**Accession codes:** ChIP-chip data have been deposited to the GEO repository under accession code GSE57358

**How to cite this article:** Naville, M. *et al*. Long-range evolutionary constraints reveal *cis*-regulatory interactions on the human X chromosome. *Nat. Commun*. 6:6904 doi: 10.1038/ncomms7904 (2015).

## Supplementary Material

Supplementary Information Supplementary Figures 1-7, Supplementary Tables 1-2 and Supplementary References

Supplementary Data 1 List of 102,647 RegHsa elements, their chromosomal positions, their linkage score and their predicted target genes.

Supplementary Data 2 List of 1,013 RegHsa elements and the results of the transgenic experiments.

Supplementary Data 3 Results of detailed comparison between transgenic GFP reporter assays and in situ mRNA patterns of predicted targets.

Supplementary Data 4 List of individual sequence instances contributing to motifs described in Figure 4 of the main text.
