# Supplementary material for: Long-range evolutionary constraints reveal cis-regulatory interactions on the human X chromosome
Source: Nat Commun. 2015 Apr 24;6:6904. doi: 10.1038/ncomms7904 (PMC4423230; doi:10.1038/ncomms7904)
Supplement: Supplementary Information — Supplementary Figures 1-7, Supplementary Tables 1-2 and Supplementary References [file ncomms7904-s1.pdf]

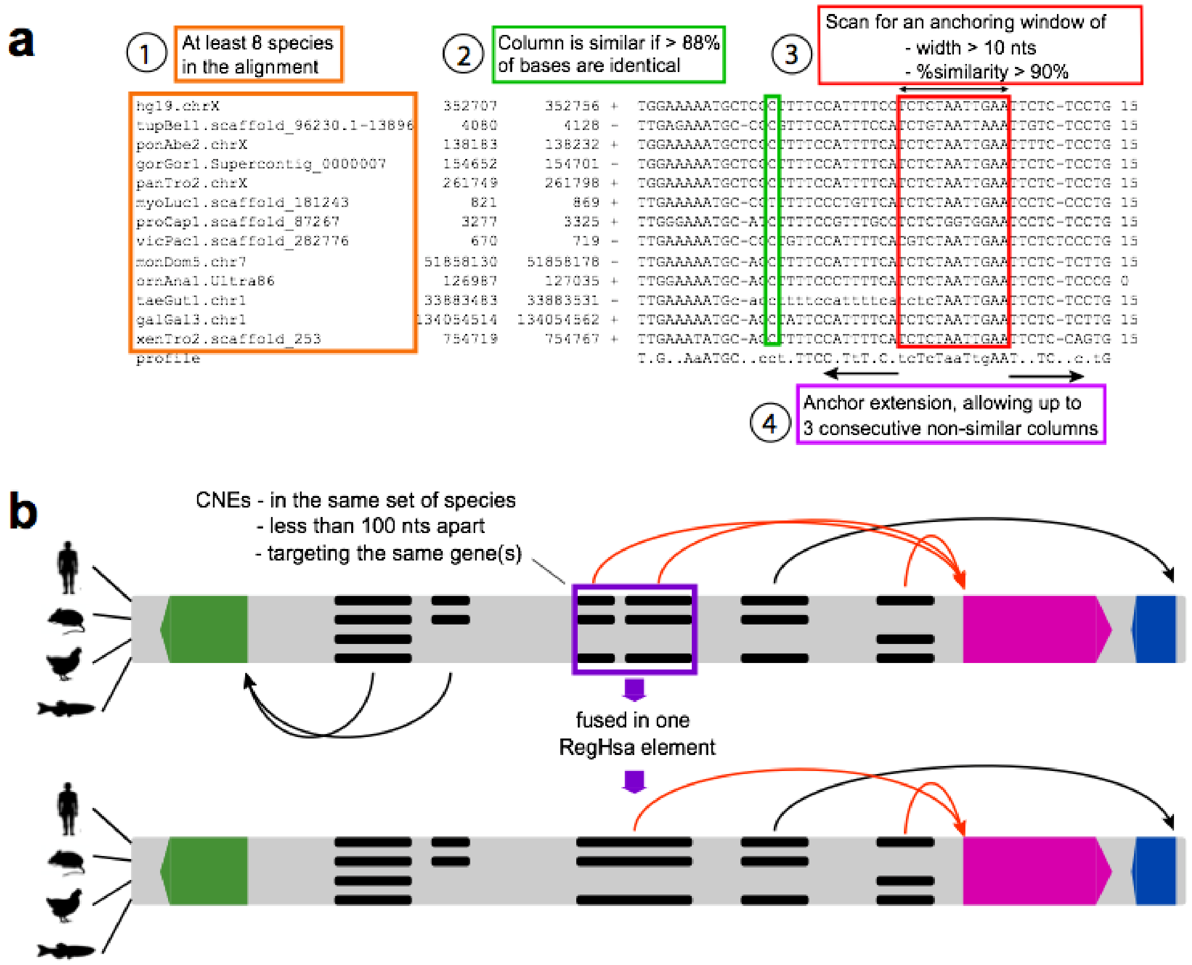

Supplementary Figure 1. Identification of Conserved Non-coding Elements and definition of REG elements.

(a) Conserved non-coding elements (CNEs) were identified in a multiple alignment of 46 vertebrate species on the human genome and available on the UCSC genome portal. Regions where at least 7 species are aligned against the human genome are scanned for a 10 bp window where at least 90% of columns are similar (contain >88% identical bases to the human base). Next, the 10 bp windows are extended on both sides as long a no more than 3 consecutive non-similar columns are encountered. **(b)** CNEs are fused in RegHsa elements if they are conserved in the same species, if they are separated by less than 100 bp in the human genome, and if they are linked with a maximal score to the same gene(s).

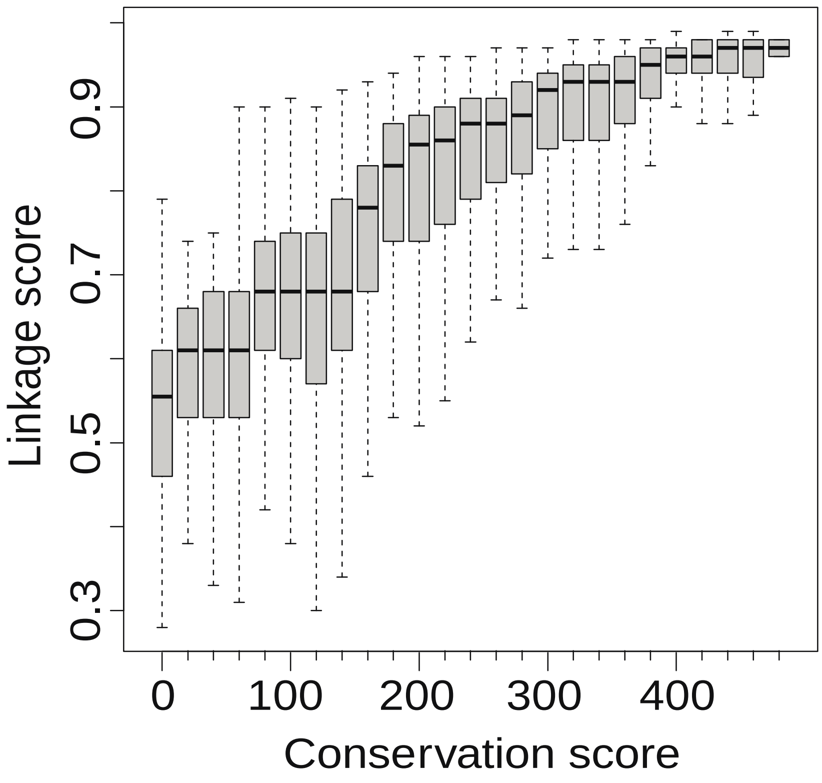

Supplementary Figure 2. Correlation between the conservation score and the linkage score. The linkage scores of the CNEs were grouped in bins according to the conservation score of the CNEs (x-axis). The distribution of the linkage score in each bin is shown as a box plot (y-axis). The median linkage score positively correlates with sequence conservation. See the methods section for details on how the conservation and the linkage score are computed.

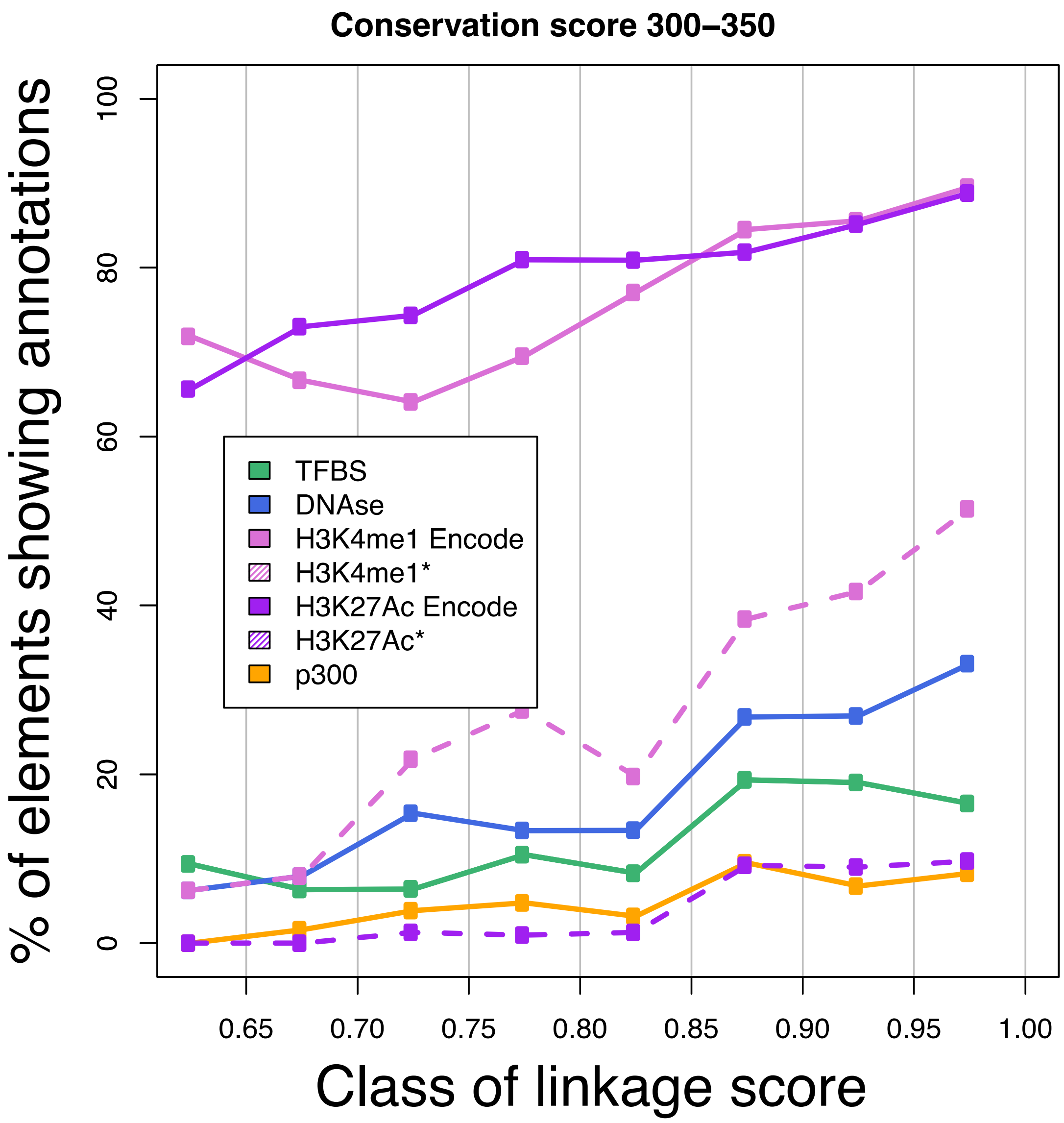

Supplementary Figure 3. RegHsa elements are enriched in functional annotations as the linkage score increases, even when controlling for conservation score. The graph shows, for different classes of RegHsa elements of increasing linkage score, the proportion of these elements that overlap annotations conventionally associated with enhancer function. Colour codes are identical to Figure 1b. Here, in contrast to Figure 1b, only RegHsas with conservation score comprised between 300 and 350 where used, showing that the enrichment in functional annotations increases even when the conservation score remains within a narrow range. An asterisk indicates data generated during this project.

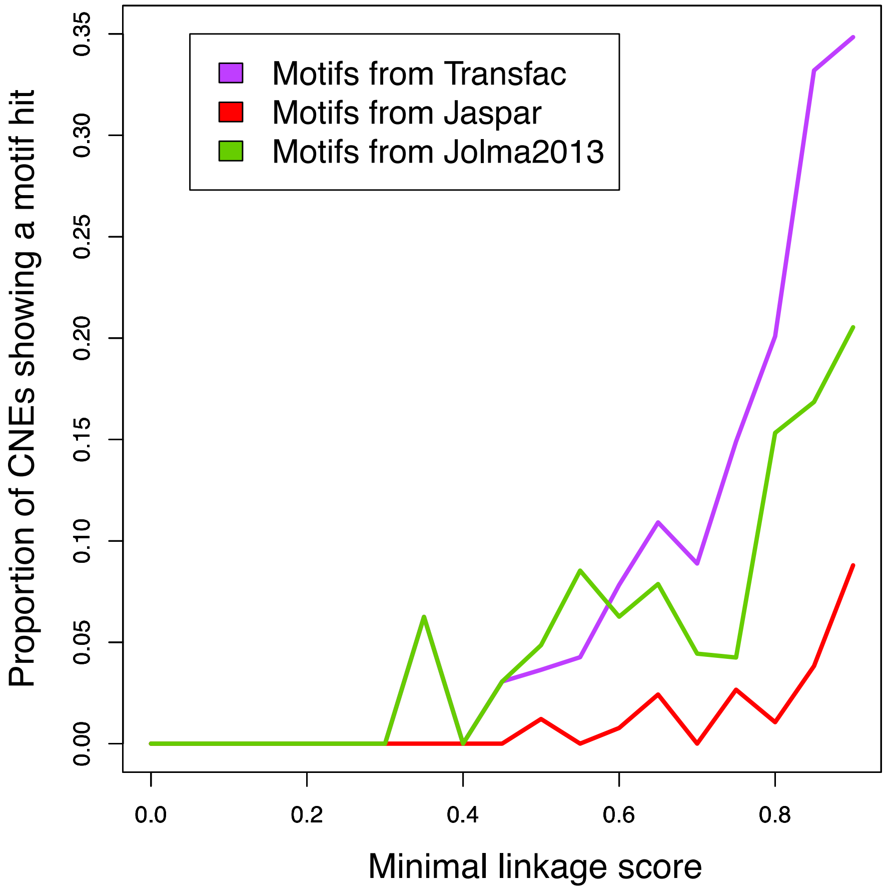

Supplementary Figure 4. Enrichment of known transcription factor binding motifs in CNEs of increasing linkage score to their target gene.

CNEs were divided in classes of increasing linkage score, and each class was compared to transcription factor binding matrices from the vertebrate component of the TRANSFAC database (version 2010)[1], to a list of matrices established by high throughput SELEX[2], and to vertebrate matrices from the Jaspar database (version 2011)[3]. See online methods for details.

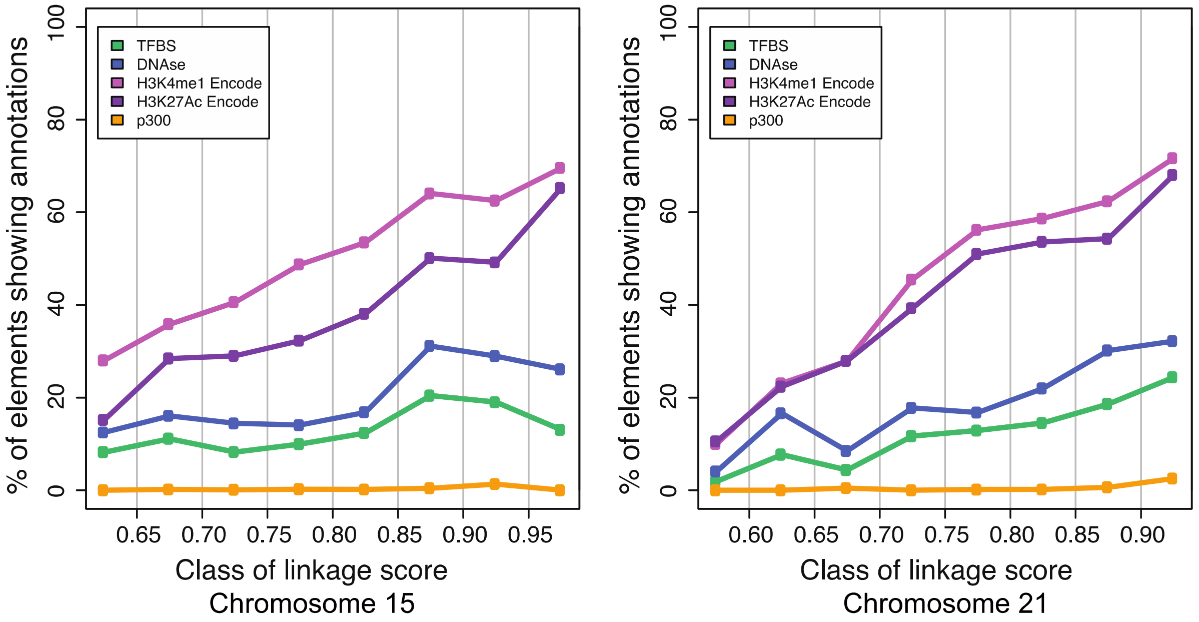

Supplementary figure 5. The linkage score was computed for chromosomes 15 and 21, and show the same enrichment in functional data conventionally associated with enhancers as shown for the X chromosome.

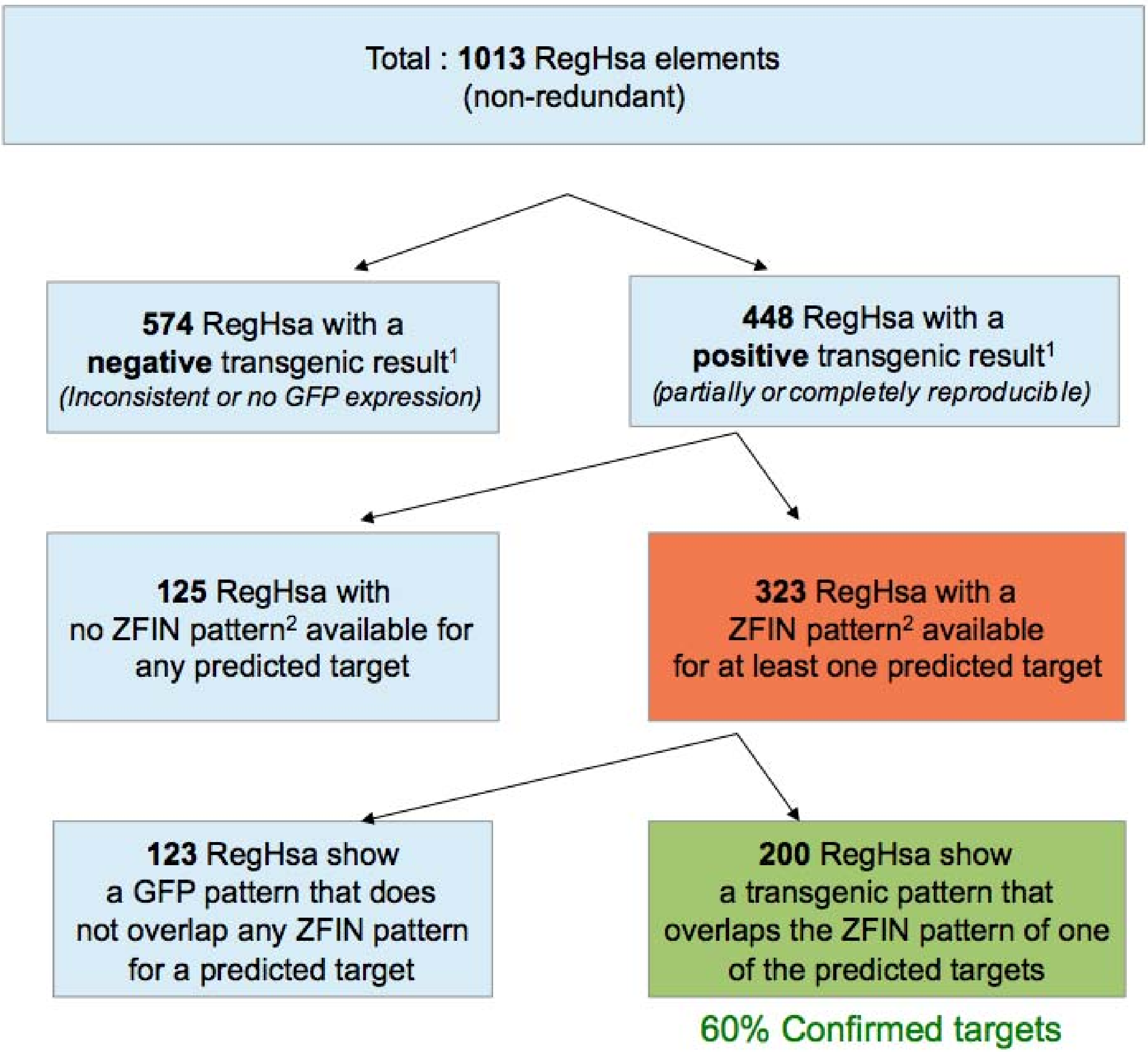

Supplementary Figure 6. Comparison between linkage score predictions and transgenic experiments.

Note 1: 574 + 448 = 1022 RegHsa elements. 9 RegHsa elements are located in 2 overlapping tested elements, one with a negative result, one with a positive result: 1022 – 9 = 1013

Note 2: ZFIN patterns are either expression of a gene measured by in situ hybridisation with a mRNA probe of the target gene, or a description of organs affected by mutation(s) in the predicted target gene.

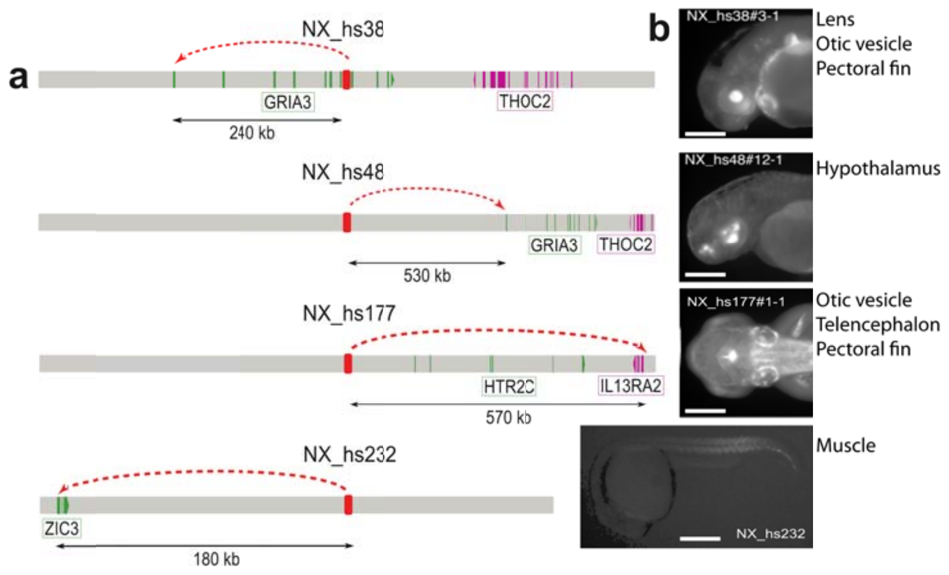

Supplementary Figure 7. Examples of long range cis-regulatory interaction predicted by evolutionary linkage score. (a) The predicted target gene is depicted in green and neighbouring genes in pink (b) The predictions are supported by transgenic analysis in zebrafish. Scale bars: NX_hs38: 200 µm; NX_hs48: 200 µm; NX_hs177: 200 µm; NX_hs232: 300 µm

Supplementary Table 1. Gene Ontology enrichment (Fisher's test) for genes linked to a RegHsa element with a score S_A > 0.9.

Name	GO ID	Total Entities	Expanded # of Entities	Overlap	Percent Overlap	Uncorrected p-value	Gene Ontology
neuronal cell body	43025	395	11	10	90	9.00E-05	cellular_component
cytosol	5829	2600	84	43	51	0.000105656	cellular_component
axon guidance	0007411, 0008040	340	16	13	81	0.000138813	biological_process
transport	0006810, 0015457, 0015460	1758	61	34	55	0.000253012	biological_process
ion channel activity	5216	334	13	11	84	0.000343556	molecular_function
catalytic activity	3824	738	17	13	76	0.000569426	molecular_function
Golgi apparatus	5794	1098	29	18	62	0.000793335	cellular_component
ATP binding	5524	1686	51	29	56	0.000864856	molecular_function
phospholipid binding	5543	374	14	11	78	0.00110333	molecular_function
nucleoplasm	5654	1033	28	17	60	0.001609	cellular_component
membrane	16020	6271	196	80	40	0.00195771	cellular_component
synapse	45202	379	18	12	66	0.00274339	cellular_component
Golgi membrane	139	505	14	10	71	0.00299736	cellular_component
ion transport	6811	629	24	15	62	0.00390496	biological_process
synapse organization	50808	35	5	5	100	0.00471183	biological_process
nucleotide binding	166	2187	87	42	48	0.00510122	molecular_function
beta-catenin binding	8013	67	5	5	100	0.00545822	molecular_function
plasma membrane	0005886, 0005904	4416	136	57	41	0.00604129	cellular_component
protein complex	43234	738	15	10	66	0.00644528	cellular_component
transmembrane transport	55085	678	25	15	60	0.00678668	biological_process
PDZ domain binding	30165	114	7	6	85	0.00935896	molecular_function
carbohydrate metabolic process	5975	417	17	11	64	0.00961903	biological_process

Supplementary Table 2. Rearrangement rates and genomic coverage used to compute the linkage score.

Species	Nb of conserved adjacencies with Homo sapiens P_e	Percent conserved adjacencies $100 \times P_e/H$	Coverage[1]	Coverage correction[2] (C_e)
Anolis carolinensis	7622	41.43	7.1	1
Bos taurus	13114	71.29	7.1	1
Callithrix jacchus	12575	68.36	6	1
Canis familiaris	12365	67.22	7.6	1
Cavia porcellus	12232	66.50	6.76	1
Choloepus hoffmanni	1197	6.50	2.05	0.34
Danio rerio	4369	23.75	6.5	1
Dasypus novemcinctus	3363	18.28	2	0.33
Dipodomys ordii	4914	26.71	1.85	0.31
Echinops telfairi	2765	15.03	2	0.33
Equus caballus	12797	69.57	6.8	1
Erinaceus europaeus	2208	12.00	1.86	0.31
Felis catus	6113	33.23	2	0.33
Takifugu rubripes	3800	20.66	8.5	1
Gallus gallus	8091	43.98	6.6	1
Gasterosteus aculeatus	4098	22.28	10.6	1
Gorilla gorilla	12255	66.62	2.1	0.35
Homo sapiens	**18393 (H)**	**100**	**10**	**1**
Loxodonta africana	12916	70.22	7	1
Macropus eugenii	1924	10.46	2	0.33
Microcebus murinus	7341	39.91	1.93	0.32
Mus musculus	13686	74.40	10	1
Monodelphis domestica	10515	57.16	6.8	1
Myotis lucifugus	10699	58.16	1.7	0.28
Ochotona princeps	6657	36.19	1.93	0.32
Ornithorhynchus anatinus	4967	27.00	6	1
Oryctolagus cuniculus	11252	61.17	7.48	1
Oryzias latipes	3693	20.07	8	1
Otolemur garnettii	5750	31.26	1.5	0.25
Pan troglodytes	15044	81.79	6	1
Pongo pygmaeus	13228	71.91	6	1
Procavia capensis	4626	25.15	2.19	0.37
Pteropus vampyrus	9153	49.76	2.63	0.44
Macaca mulatta	12589	68.44	5.1	0.85
Rattus norvegicus	11802	64.16	3	0.5
Sorex araneus	2414	13.12	1.9	0.32
Spermophilus tridecemlineatus	4981	27.08	1.9	0.32
Taeniopygia guttata	7574	41.17	6	1
Tarsius syrichta	1653	8.98	1.82	0.3
Tetraodon nigroviridis	3068	16.68	7.9	1
Tupaia belangeri	5211	28.33	2	0.33
Tursiops truncatus	9287	50.49	2.59	0.43
Vicugna pacos	5109	27.77	2.51	0.42
Xenopus tropicalis	6492	35.29	7.65	1
Petromyzon marinus	291	1.58	5	0.83

Note 1: The coverage is taken from the relevant genome project information. For human and mouse it is arbitrarily set to 10X.

Note 2: The correction is applied to compensate for assemblies where the low sequence coverage leads to small scaffolds, causing excessive interruptions in the continuity of genes along chromosomes. The contribution of assemblies with a sequence coverage C < 6X is down weighted by a factor C/6.

Supplementary References

1. Wingender, E. *et al.* The TRANSFAC system on gene expression regulation. *Nucleic Acids Res* **29**, 281-283. (2001).
2. Jolma, A. *et al.* DNA-binding specificities of human transcription factors. *Cell* **152**, 327-339 (2013).
3. Mathelier, A. *et al.* JASPAR 2014: an extensively expanded and updated open-access database of transcription factor binding profiles. *Nucleic Acids Res* **42**, D142-D147 (2014).
